# Hydrophilic Interaction Liquid Chromatography–Hydrogen/Deuterium Exchange–Mass Spectrometry (HILIC-HDX-MS) for Untargeted Metabolomics

**DOI:** 10.3390/ijms25052899

**Published:** 2024-03-01

**Authors:** Tomas Cajka, Jiri Hricko, Stanislava Rakusanova, Kristyna Brejchova, Michaela Novakova, Lucie Rudl Kulhava, Veronika Hola, Michaela Paucova, Oliver Fiehn, Ondrej Kuda

**Affiliations:** 1 Institute of Physiology of the Czech Academy of Sciences, Videnska 1083, 14200 Prague, Czech Republic; jiri.hricko@fgu.cas.cz (J.H.); stanislava.rakusanova@fgu.cas.cz (S.R.); kristyna.brejchova@fgu.cas.cz (K.B.); michaela.novakova@fgu.cas.cz (M.N.); lucie.kulhava@fgu.cas.cz (L.R.K.); veronika.hola@fgu.cas.cz (V.H.); michaela.paucova@fgu.cas.cz (M.P.); ondrej.kuda@fgu.cas.cz (O.K.); 2 West Coast Metabolomics Center, University of California, Davis, 451 Health Sciences Drive, Davis, CA 95616, USA; ofiehn@ucdavis.edu

**Keywords:** metabolomics, liquid chromatography, mass spectrometry, hydrogen/deuterium exchange, unknown identification

## Abstract

Liquid chromatography with mass spectrometry (LC-MS)-based metabolomics detects thousands of molecular features (retention time–*m*/*z* pairs) in biological samples per analysis, yet the metabolite annotation rate remains low, with 90% of signals classified as unknowns. To enhance the metabolite annotation rates, researchers employ tandem mass spectral libraries and challenging in silico fragmentation software. Hydrogen/deuterium exchange mass spectrometry (HDX-MS) may offer an additional layer of structural information in untargeted metabolomics, especially for identifying specific unidentified metabolites that are revealed to be statistically significant. Here, we investigate the potential of hydrophilic interaction liquid chromatography (HILIC)-HDX-MS in untargeted metabolomics. Specifically, we evaluate the effectiveness of two approaches using hypothetical targets: the post-column addition of deuterium oxide (D_2_O) and the on-column HILIC-HDX-MS method. To illustrate the practical application of HILIC-HDX-MS, we apply this methodology using the in silico fragmentation software MS-FINDER to an unknown compound detected in various biological samples, including plasma, serum, tissues, and feces during HILIC-MS profiling, subsequently identified as *N*^1^-acetylspermidine.

## 1. Introduction

Metabolomics studies low-molecular-weight compounds, typically <2000 Da, within complex biological systems under specific conditions [1]. For the analysis of polar metabolites, liquid chromatography with mass spectrometry (LC-MS), specifically reversed-phase liquid chromatography (RPLC) and hydrophilic interaction liquid chromatography (HILIC), serves as the primary separation technique [2], followed by gas chromatography (GC) [3] and capillary electrophoresis (CE) [4,5]. Overall, LC-MS has become the most applied chromatography–MS tool for analyzing polar and nonpolar metabolites such as lipids [4]. Indeed, a combination of multiple chromatography–MS-based platforms has become a common approach to achieving wide metabolome and lipidome coverage [6,7,8,9].

LC-MS-based metabolomics can generate thousands of molecular features/ion peaks (i.e., retention time–*m*/*z* pairs) in biological samples per single analysis. However, it is estimated that only 10% of these features can be annotated according to mass spectra library matches [10,11], leaving the remaining 90% as unknowns. Thus, metabolomics faces a low identification rate [12] despite the overcounting of features that either do not trigger the accompanying MS/MS spectra or need to be accounted for differently due to adducts [13,14]. Untargeted metabolomics studies often provide only sets of annotated metabolites, unknowns are not further investigated because additional resources are needed, and the structure elucidation process is time-consuming. However, studies focused, for instance, on circadian rhythms [12,15], cardiovascular diseases [16], type 2 diabetes [17], lung cancer [18], or nutrition [19] have shown that these unknowns can be statistically significant, and focus on their structure elucidation may bring valuable insights into metabolic pathways, potentially uncovering novel biomarkers or therapeutic targets associated with these specific physiological and pathological conditions [20].

The metabolite annotation rate can be increased by collecting information from the MS analysis of certified standards and making these data publicly accessible in mass spectral repositories [21,22]. However, acquiring the MS/MS spectra of all authentic metabolites in every laboratory is unrealistic. As a more viable solution, tandem mass spectral libraries were developed to annotate the MS/MS spectra obtained during metabolomics experiments. Despite the availability of various commercial and public MS/MS libraries such as NIST 23, METLIN Gen2, MassBank, and MoNA [21,23,24], many false positive annotations may be reported [9,25,26].

Employing computational simulations to predict the mass spectra from input structures can further increase the annotation rate [27]. Various existing in silico fragmentation software programs, including Mass Frontier, CSI:FingerID, CFM-ID, MS-FINDER, MIDAS-G, and MetFrag [27,28,29,30,31,32], are utilized for identifying unknown metabolites and determining their chemical structures. Typically, these tools convert mass data into molecular fragments using combinatorial structure generation techniques with limited success. Hence, it is crucial to integrate additional orthogonal information to determine the correct structure. Such methods may be used to annotate unknown compounds while also reducing false positive annotations originating from MS/MS search and in silico fragmentation software. Hydrogen/deuterium exchange mass spectrometry (HDX-MS), a well-established technique in proteomics and for the structural elucidation of pharmaceutical impurities and drug metabolites, though not fully explored in metabolomics, provides a promising approach [33,34,35,36,37,38,39,40,41,42,43].

Exchangeable hydrogen atoms (also called active, acidic, or labile), bound to heteroatoms such as oxygen, nitrogen, and sulfur, readily exchange with deuterium, while those bound to carbon remain unaltered [44]. When metabolites are exposed to deuterium oxide (D_2_O), labile hydrogens within various functional groups such as −NH−, −NH_2_, −OH, −COOH, and −SH are substituted with deuterium. Using a mass spectrometer, the number of replaced hydrogens in the molecule can be determined by measuring the molecular mass before and after HDX. Such information can be a valuable filter, narrowing down potential candidates for structure elucidation [45,46].

Notably, HDX-MS has been successfully used to identify compounds like *N*,*N*,*N*-trimethyl-l-alanyl-l-proline betaine [47] and (2*R*,3*R*)-2,3-dihydroxy-5-methylthio-4-pentenoic acid [48] in human plasma, endogenous carbonyl steroids in human serum [49], complex lipids in mouse brains [50], secondary metabolites in root exudates of *Arabidopsis thaliana* [51], and *N*-methyl lysophosphatidylethanolamines as abundant lipids in acidophilic mixed microbial communities [34]. However, these studies did not extensively cover the development and overall performance of the HDX approach.

Here, we evaluate the potential of LC-HDX-MS in untargeted metabolomics. Specifically, we compare the performance of two methods: the post-column addition of D_2_O and the on-column HILIC-HDX-MS method. In the latter case, we also evaluate variations when only water is replaced with D_2_O and non-deuterated mobile-phase modifiers are used (partial HILIC-HDX-MS) or both D_2_O and deuterated mobile-phase modifiers are used (full HILIC-HDX-MS). We exemplify this approach by identifying an unknown metabolite detected in various biological samples (biofluids, tissues, feces) of rats, mice, and humans.

## 2. Results and Discussion

Untargeted metabolomics and lipidomics studies typically report hundreds of annotated metabolites, usually after combining data from various LC-MS platforms. However, many more metabolites remain unidentified. Elemental formulas can be correctly assigned in 95% of all unknowns [9], but annotating these compounds is challenging. Chemical transformations such as HDX may reveal substructure information and be used to discard false positive isomer structures from chemical database queries to differentiate lists of possible isomers.

For this evaluation, we adapted our fast HILIC-MS method [52], utilizing an ACQUITY Premier BEH Amide column (50 mm × 2.1 mm i.d.; 1.7 μm particle size) equipped with a VanGuard FIT cartridge (5 mm × 2.1 mm i.d.; 1.7 μm particle size) and employing an 8.5 min injection-to-injection time. The HILIC-MS method uses acetonitrile/water (95:5) and water as mobile phases, both with ammonium formate (10 mM) and formic acid (0.125%) (Table 1).

**Figure 1:**
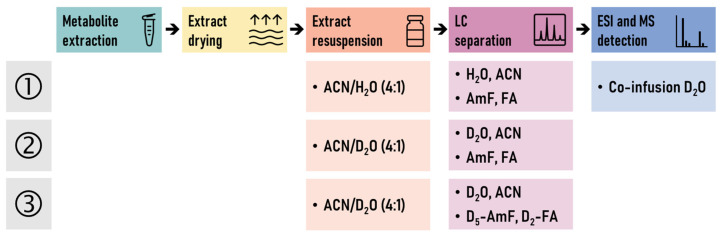
Experimental design of LC-HDX-MS experiments: setup (1) of conventional HILIC-MS method with a post-column addition of D_2_O, setup (2) of partial on-column LC-HDX-MS method, and setup (3) of full on-column LC-HDX-MS method. ACN, acetonitrile; AmF, ammonium formate; FA, formic acid.

Considering the simplicity of operation, we initially focused on evaluating the performance of the standard HILIC-MS profiling method, modified only with the post-column addition of D_2_O. In this case, the previously injected extracts in an acetonitrile/water (4:1, *v*/*v*) mixture were subsequently analyzed using the same HILIC-MS method but with a post-column addition of D_2_O introduced using an infusion pump (Figure 1, Table 1). The advantage of this approach is that no further modification of the mobile phases and resuspended extracts was needed.

Next, we turned our attention to on-column HILIC-HDX-MS methods with modified mobile phases and the resuspension of dry extracts. For partial HILIC-HDX-MS, only water was replaced by D_2_O, and non-deuterated mobile-phase modifiers (ammonium formate and formic acid) were used. The dry extracts were resuspended in an acetonitrile/D_2_O (4:1, *v*/*v*) mixture. The advantage of this approach is that only the key H/D exchange solvent (D_2_O) was needed. On the other hand, for full HILIC-HDX-MS, both D_2_O and deuterated mobile-phase modifiers (D_5_-ammonium formate and D_2_-formic acid) were employed, and the dry extracts were again resuspended in an acetonitrile/D_2_O (4:1, *v/v*) mixture (Figure 1, Table 1). All the H/D exchange components were replaced in this last tested method, thus requiring more individual D-labeled chemicals during the run.

### 2.1. HILIC-MS with the Post-Column Addition of D_2_O

**Figure 2:**
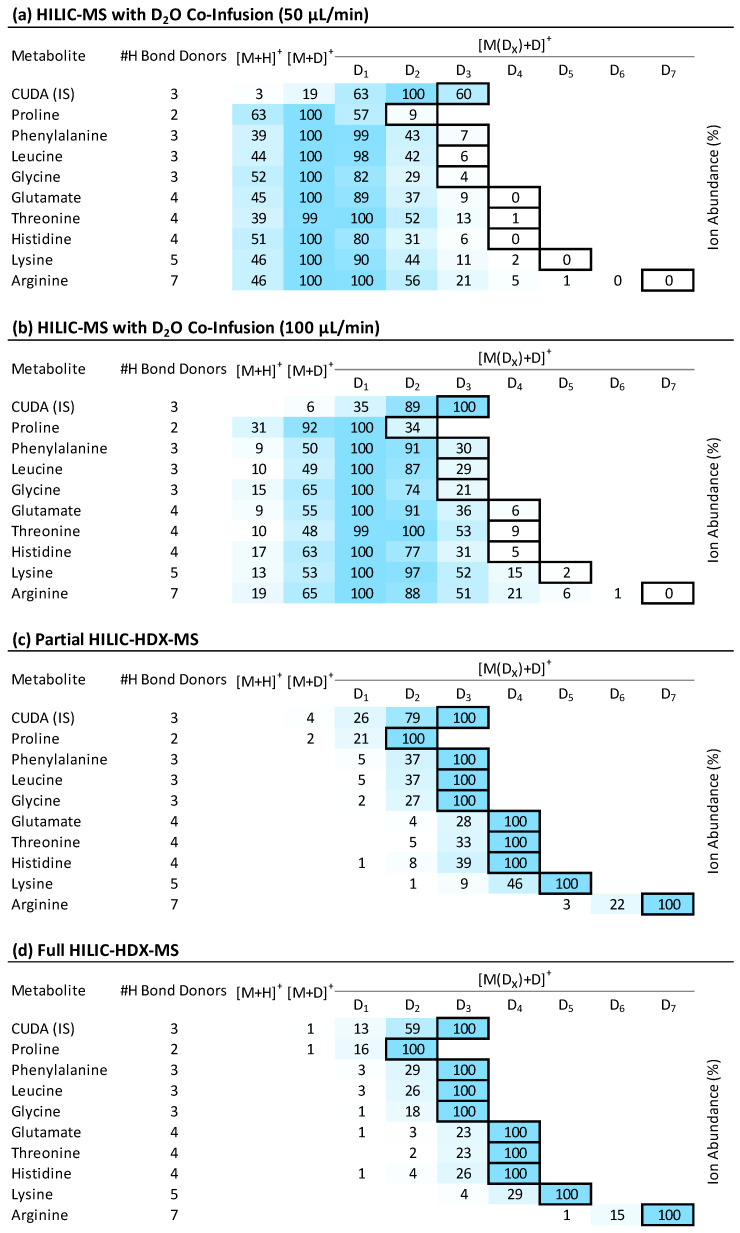
Evaluation of selected target compounds containing 2–7 labile hydrogens (#H Bond Donors) under (**a**) conventional HILIC-MS with post-column addition of D_2_O (50 µL/min); (**b**) conventional HILIC-MS with post-column addition of D_2_O (100 µL/min); (**c**) partial HILIC-HDX-MS with D_2_O used in the mobile phases; (**d**) full HILIC-HDX-MS with D_2_O, D_5_-ammonium formate, D_2_-formic acid used in the mobile phases. Full H/D exchange is indicated with a solid box (□). The most abundant ion (base peak) in the MS1 spectrum is labeled blue (■), and the color of lower intensities is proportionally scaled. For details of the composition of mobile phases and used modifiers, see Table 1.

**Figure 3:**
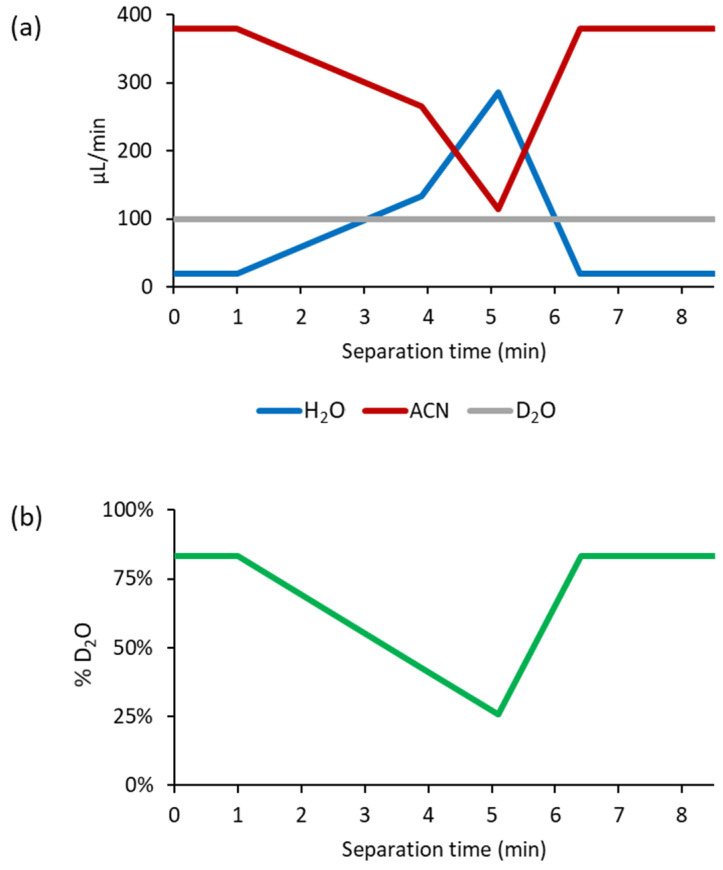
(**a**) Total flow of H_2_O and acetonitrile (ACN) from mobile phase A (H_2_O) and mobile phase B (acetonitrile/H_2_O 95:5) and post-column addition of D_2_O (100 µL/min). (**b**) Ratio of D_2_O/(D_2_O+H_2_O) when co-infusing D_2_O (100 µL/min) during the HILIC method (400 µL/min).

For the first setup, we used the post-column addition of D_2_O introduced via a Tee connector into the LC effluent using an infusion pump. We first evaluated this approach for a range of polar metabolites with varying numbers of exchangeable hydrogen atoms. For these hypothetical targets, we assessed the extent of H/D exchange. As Figure 2a shows, the post-column co-infusion of D_2_O at a flow rate of 50 µL/min against a HILIC column flow of 400 µL/min led to the detection of the molecules as [M+D]^+^ ions as base peaks (100%), and only limited H/D exchange occurred. A slight improvement was observed with the increased addition of D_2_O (100 µL/min), leading to the detection of base peaks for [M(D_1_)+D]^+^ ions with one H/D exchange (Figure 2b). We also included in Figure 2 an internal standard, 12-[[(cyclohexylamino)carbonyl]amino]-dodecanoic acid (CUDA), eluting at 0.45 min, in which case the co-infusion of D_2_O led to complete H/D exchange ([M(D_3_)+D]^+^). This can be explained by the low content of H_2_O (Figure 3a) in the mobile phase with co-infused D_2_O (~83% D_2_O, Figure 3b).

However, most polar metabolites were eluted during HILIC-MS at the composition of the mobile phases, favoring a high content of H_2_O (3–5 min); thus, the content of co-infused D_2_O (100 µL/min) is reduced to 25–40% compared to H_2_O from the mobile phases (Figure 3b). This shortcoming led to the incomplete H/D exchange mass spectra of the evaluated polar metabolites, which would be difficult to interpret if these were unknowns. Shah et al. [37] showed with the example of drug metabolites that the post-column addition of D_2_O was efficient when the LC output was 50 µL/min and a D_2_O infusion rate of 126 µL/min (~1:2.5 ratio) was used. However, splitting the LC flow was required, possibly leading to a signal reduction and requiring further modification of the LC-MS setup. Of note, resuspending the samples in an acetonitrile/D_2_O (4:1, *v*/*v*) mixture with follow-up analysis did not improve the H/D exchange. Thus, the post-column addition of D_2_O appeared to provide limited potential for H/D exchange, as also highlighted by Liu et al. [33] during pharmaceutical compound analysis.

### 2.2. Partial and Full HILIC-HDX-MS

In the subsequent two setups of on-column HILIC-HDX-MS, we replaced H_2_O with D_2_O as the component of both mobile phases but kept unlabeled mobile-phase modifiers (ammonium formate, formic acid), i.e., partial HILIC-HDX-MS, or also replaced the mobile-phase modifiers with deuterated counterparts (D_5_-ammonium formate, D_2_-formic acid), i.e., full HILIC-HDX-MS.

However, for mobile phase B containing acetonitrile and D_2_O (95:5, *v/v*), we observed solubility issues with both 10 mM ammonium formate and 10 mM D_5_-ammonium formate compared to the acetonitrile/H_2_O (95:5, *v*/*v*) mixture with 10 mM ammonium formate. Based on our experience, the light turbidity disappears after sonication when preparing fully non-labeled mobile phase B. However, this was not the case when D_2_O was used instead of H_2_O. For complete dissolving of the salts, sonication at 25 °C for 10 mM ammonium formate or even 30 °C for the D_5_-ammonium formate solutions was needed.

Unfortunately, after cooling both solutions to lab temperature (23 °C), we observed crystals of the salts, although the solutions were not turbid. We found that 7.5 mM of ammonium formate or D_5_-ammonium formate was a limiting concentration when D_2_O was used as a part of mobile phase B. However, this modification of mobile phase B did not impact the metabolites’ retention time shift or peak shape.

Figure 2c,d show that the partial and full HILIC-HDX-MS setups provided complete H/D exchange for the evaluated metabolites compared to the setup with the post-column addition of D_2_O. The full HILIC-HDX-MS method demonstrated slightly better performance, as the intensities of the [M(D*_x_*_–1_)+D]^+^ ions were lower (with an average absolute difference of ~10%) compared to the [M(D*_x_*)+D]^+^ ions, which represent the full H/D exchange species with *x* deuterium atoms. The increased ratio of [M(D*_x_*_–1_)+D]^+^/[M(D*_x_*)+D]^+^ ions in the partial HILIC-HDX-MS method can be attributed to the presence of non-deuterated mobile-phase modifiers providing hydrogen atoms, thereby reducing the yield of fully deuterated ions.

We should also note that the H/D exchange efficiency has been reported to be pH-dependent [53]. For example, amides demonstrate a minimum exchange rate around a pH of 2.5 [54], while carbohydrates, primarily composed of hydroxyls, exhibit a minimum exchange rate around a pH of 6.5, with the exchange rate increasing as the solution becomes more acidic or basic [55]. The pH of the mobile phases used in the HILIC method was approximately 3, aligning with the observed pH-dependent trends in amides and carbohydrates. However, as metabolomic profiling encompasses diverse groups of metabolites, the H/D exchange efficiency can vary based on the pH of the mobile phase.

**Figure 4:**
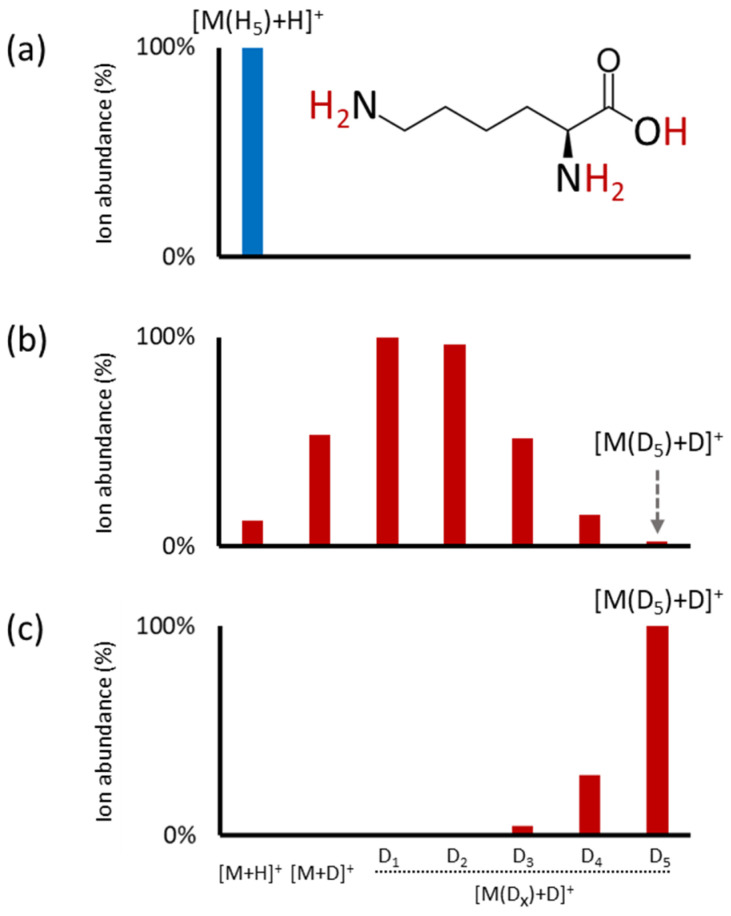
MS1 spectra of amino acid lysine obtained using (**a**) conventional HILIC-MS (*m*/*z* 147.1128, [M+H]^+^), (**b**) conventional HILIC-MS with post-column addition of D_2_O (100 µL/min), and (**c**) full HILIC-HDX-MS (*m*/*z* 153.15046, [M(D_5_)+D]^+^). The lysine structure indicates the molecule’s possible exchangeable hydrogens (in red).

Figure 4 shows an example of the amino acid lysine with five labile hydrogen atoms acquired using conventional HILIC-MS with detected [M+H]^+^ ions (Figure 4a), followed by incomplete H/D exchange when using the post-column addition of D_2_O, providing ions from [M+H]^+^ to [M(D_5_)+D]^+^ (Figure 4b), and complete H/D exchange when the full HILIC-HDX-MS method was used, with dominating [M(D_5_)+D]^+^ ions (Figure 4c). All these data show that on-column H/D exchange is advantageous since it generally produces a high yield of fully deuterated compounds owing to the adequate mixing time of the metabolites with the deuterated mobile phases.

Regarding D_2_O consumption, 850 µL of D_2_O per injection was necessary when utilizing the post-column addition of D_2_O (100 µL/min), whereas approximately 600 µL of mobile phase A with D_2_O and 2.8 mL of mobile phase B (including ~140 µL D_2_O) was required for the partial or full HILIC-HDX-MS setups. Thus, both approaches involved comparable volumes of D_2_O. However, the partial or full HILIC-HDX-MS setups provided more efficient deuterium incorporation into the analytes, making them the preferred choice for achieving complete H/D exchange in untargeted metabolomics studies.

### 2.3. Structure Elucidation of N^1^-Acetylspermidine Using HILIC-HDX-MS

When processing raw HILIC-MS files from multiple studies focused on nutritional intervention [56], circadian rhythms [57], drug treatment [58], or heart failure [59], we have repeatedly observed an unknown metabolite eluted at a retention time of 4.3 min and *m*/*z* 188.1757 in various biological matrices (biofluids, tissues, feces) of rats, mice, and humans (Figure S1).

Since we did not obtain a positive spectral match when using the combined NIST 23 and MoNA MS/MS libraries, we submitted MS1 isotopic ions and the MS/MS spectrum from MS-DIAL to the MS-FINDER software [27] for structure elucidation. Only one formula (C_9_H_21_N_3_O), within a mass error of <0.005 Da (set up as a criterion for mass tolerance), was reported.

This formula provided the source to 23 local databases in MS-FINDER with 101 possible unique structures. Focusing on such a high number of potential candidates would be impractical; thus, we applied an additional filter from the HILIC-HDX-MS experiment. Specifically, we obtained information based on the isotopic ions from the conventional HILIC-MS used for polar metabolite profiling and full HILIC-HDX-MS that four labile hydrogens were present in the molecule. Using this information, we reduced the number of potential candidates to 22; thus, 78% of false positive structures were filtered out with this additional filter.

**Figure 5:**
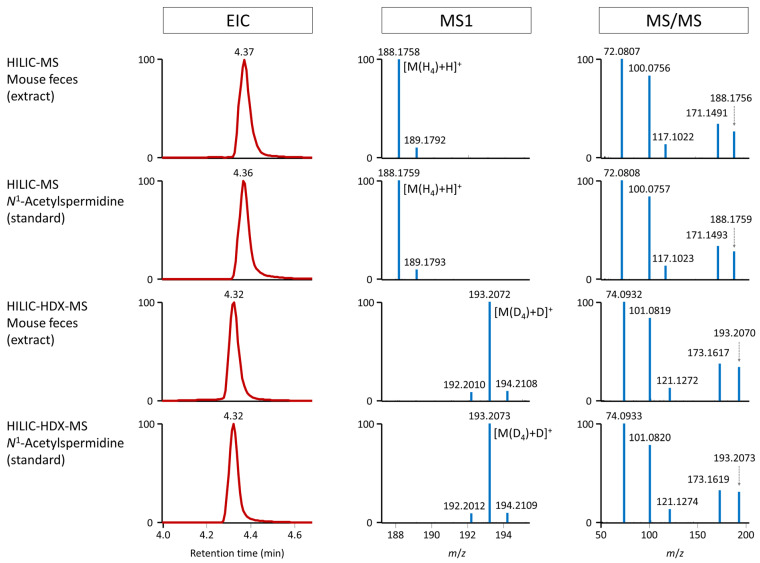
Extracted ion chromatograms (EICs) and MS1 and MS/MS spectra of *N*^1^-acetylspermidine in mouse feces and analytical standard analysis under conventional HILIC-MS and full HILIC-HDX-MS conditions. In HILIC-MS, the EIC at *m*/*z* 188.1757, corresponding to [M(H_4_)+H]^+^, is displayed. Conversely, in HILIC-HDX-MS, the EIC at *m*/*z* 193.2071, corresponding to [M(D_4_)+D]^+^, is shown. MS/MS spectra were acquired at stepped normalized collision energies of 20, 30, and 40%.

**Figure 6:**
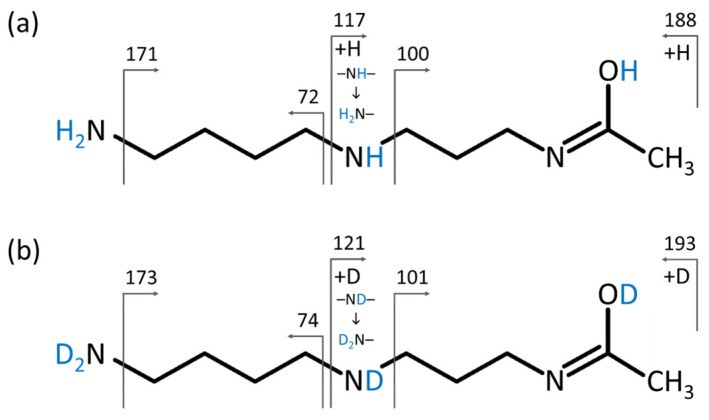
MS/MS fragmentation of *N*^1^-acetylspermidine under (**a**) HILIC-MS (precursor ion *m*/*z* 188.1757, [M(H_4_)+H]^+^) and (**b**) full HILIC-HDX-MS (precursor ion *m*/*z* 193.2071, [M(D_4_)+D]^+^) conditions.

Based on this information and using the two most scored candidates, we analyzed the standards of *N*^1^-acetylspermidine and *N*^8^-acetylspermidine using HILIC-MS and HILIC-HDX-MS. Figure 5 shows that the *N*^1^-acetylspermidine detected in mouse feces matched the standard, including the retention time and MS1 and MS/MS spectra from HILIC-MS and HILIC-HDX-MS after H/D exchange. A similar observation was also confirmed for rat feces (Figure S2) and human plasma NIST SRM 1950 (Figure S3). Figure 6 also shows the MS/MS fragmentation of *N*^1^-acetylspermidine under HILIC-MS and full HILIC-HDX-MS conditions, further confirming the identity of this unknown metabolite based on H/D exchange in a series of MS/MS fragments. Of note, the standard of *N*^8^-acetylspermidine provided a longer retention time (0.05 min shift) than the peak of *N*^1^-acetylspermidine detected in mouse feces, and the MS/MS spectrum did not match (Figure S4). The MS/MS spectra of *N*^1^-acetylspermidine and *N*^8^-acetylspermidine can be downloaded in a mass searchable format (MSP) for storing MS/MS spectra (*m*/*z* and intensity of mass peaks) from the Supplementary Materials.

**Figure 7:**
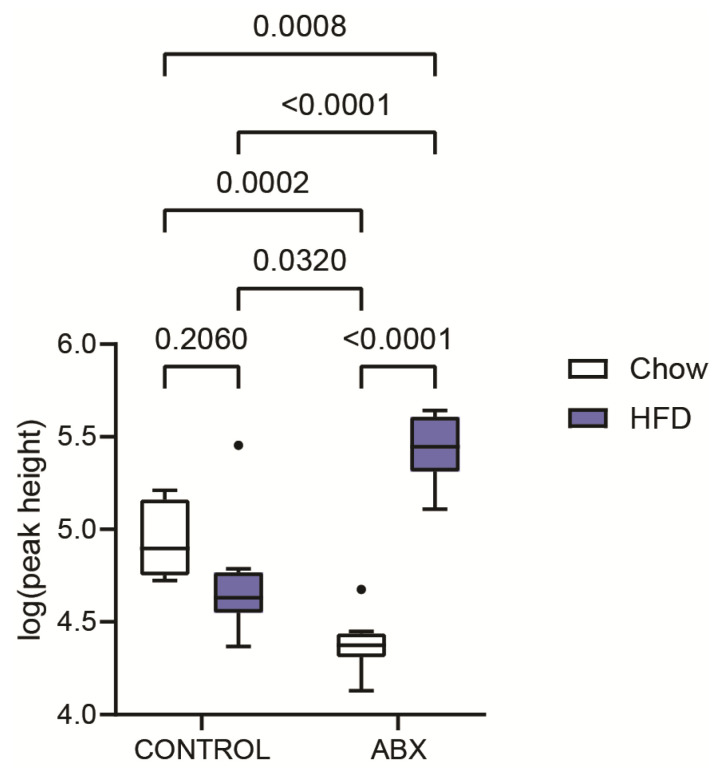
Box plots of *N*^1^-acetylspermidine during experiments investigating the impact of diet (chow vs. high-fat diet) and antibiotic (ABX) treatment on mice. For statistical analysis, two-way ANOVA was used.

In addition, *N*^1^-acetylspermidine appeared statistically significant during our experiments investigating the impact of diet and antibiotic treatment on mice. As Figure 7 shows, there was no statistical difference between the groups of mouse feces under normal (chow) and high-fat diets. On the other hand, after antibiotic treatment, over one order of magnitude difference in signal intensity was observed between these two groups. Interestingly, in mice on a chow diet, the level of *N*^1^-acetylspermidine decreased 3.8-fold after antibiotics treatment, while on a high-fat diet, the opposite trend was observed (4.1-fold increase), indicating differences in the polyamine pathway of the microbiota.

## 3. Materials and Methods

### 3.1. Materials and Reagents

For the sample extraction, methanol (J.T.Baker, Phillipsburg, NJ, USA, catalog no. 9822), methyl *tert*-butyl ether (Honeywell, Charlotte, NC, USA, catalog no. 34875), and water (VWR, Suwanee, GA, USA, catalog no. 83645.320) were used. The LC-MS-grade solvents for mobile phases included acetonitrile (Honeywell, catalog no. 34967) and water (VWR, catalog no. 83645.320). Mobile-phase modifiers such as ammonium formate (Supelco, Bellefonte, PA, USA, catalog no. 70221) and formic acid (VWR, catalog no. 84865.260) were also of LC-MS-grade quality. The deuterium oxide (catalog no. 617385) and D_5_-ammonium formate (catalog no. 795119) were from Merck, Rahway, NJ, USA. The D_2_-formic acid (catalog no. DLM-286-PK) was from Cambridge Isotope Laboratories, Tewksbury, MA, USA. The *N*^1^-acetylspermidine (catalog no. 9001535; InChI Key: MQTAVJHICJWXBR-UHFFFAOYSA-N) and *N*^8^-acetylspermidine (catalog no. 37434; InChI Key: FONIWJIDLJEJTL-UHFFFAOYSA-N) were from Cayman Chemical, Tallinn, Estonia.

The mouse and rat feces samples were from the Institute of Physiology of the Czech Academy of Sciences, Prague, Czech Republic. The human serum (catalog no. S7023-100ML), NIST SRM 1950 plasma (catalog no. NIST1950), and a mixture of 17 amino acids (catalog no. 79248) were from Merck.

### 3.2. Experiments with Animals

The age-matched 6-week-old male C57BL/6J mice were from Charles River Laboratories (Sulzfeld, Germany). After their arrival, the mice were individually housed in cages and maintained at 22 °C and according to a 12 h light/dark cycle (light from 6:00 a.m.). The mice were maintained on a chow or high-fat diet (ssniff Spezialdiäten, Soest, Germany) ad libitum. The chow diet contained 16% calories from fat, 27% from protein, and 57% from carbohydrates. The high-fat diet contained 60% calories from fat, 20% from protein, and 20% from carbohydrates. One group of mice was on a chow diet (*n* = 16) for three weeks prior to the subsequent experiments, while the other group of mice (*n* = 16) was first on a chow diet for one week (acclimatization), followed by two weeks on a high-fat diet. After these initial periods, each group was split into (i) a control group (no antibiotics) (*n* = 8) and (ii) a treatment group (with antibiotics) (*n* = 8) for two weeks. Ampicillin, streptomycin, and clindamycin at a ratio of 1:1:1 were provided in sterile drinking water at a final concentration of 1 g/L. These antibiotics were chosen due to their broad spectrum capacity and well-documented impacts on the intestinal microbiota [60]. The animals were allowed to drink ad libitum during the experiment, with water replacement at 3-day intervals. Feces samples were collected and stored at −80 °C until further analysis.

### 3.3. Sample Preparation

The metabolites were extracted using a biphasic solvent system of cold methanol, methyl *tert*-butyl ether, and water [52,61].

For the extraction of biofluids (plasma, serum), a 25 µL aliquot in a 1.5 mL tube was shaken (30 s) with 165 µL ice-cold methanol and 600 µL MTBE, both containing internal standards [61]. Subsequently, 165 µL of 10% methanol with internal standards [61] was added, vortexed (10 s), and then centrifuged (24,328× *g*, 5 min, 4 °C). A 70 µL aliquot of the bottom phase was collected and evaporated. The dry extracts were resuspended in 70 µL of an acetonitrile/water (4:1) or acetonitrile/D_2_O (4:1) mixture with two internal standards (CUDA and Val-Tyr-Val) based on the experimental setup tested (see Table 1). After shaking (30 s), the samples were centrifuged (24,328× *g*, 5 min, 4 °C) and analyzed using the HILIC metabolomics platform [52].

For the extraction of tissues and feces, 20 mg of the sample in a 2 mL tube was homogenized (1.5 min) with 275 μL of methanol using a grinder. Subsequently, 1 mL of MTBE was added, and this mixture was shaken (30 s). Finally, 275 μL of 10% methanol was added, and after vortexing (10 s), the tubes were centrifuged (24,328× *g*, 5 min, 4 °C) [61]. A 70 µL aliquot of the bottom phase was collected and evaporated. The dry extracts were further processed as described for biofluids.

### 3.4. LC-MS Conditions

The LC-MS system comprised a Vanquish UHPLC system (Thermo Fisher Scientific, Bremen, Germany) and a Q Exactive Plus mass spectrometer (Thermo Fisher Scientific) equipped with a heated electrospray ionization (HESI-II) probe (Thermo Fisher Scientific), [52,61].

The ACQUITY Premier BEH Amide column (50 mm × 2.1 mm i.d.; 1.7 µm particle size) equipped with a VanGuard FIT cartridge (5 mm × 2.1 mm i.d.; 1.7 µm particle size) (Waters, Milford, MA, USA) was used to separate the polar metabolites. The column was maintained at 45 °C. The composition of the mobile phases, resuspension solvent, and column flow rate are summarized in Table 1. Separation was conducted under the following gradient: 0 min 100% (B); 0–1 min 100% (B); 1–3.9 min from 100% to 70% (B); 3.9–5.1 min from 70% to 30% (B); 5.1–6.4 min from 30% to 100% (B); 6.4–7.5 min 100% (B) +1 min pre-injection steps. An injection volume of 1 μL was used. The sample temperature was maintained at 4 °C.

The ESI source and MS settings were as follows: sheath gas pressure, 50 arbitrary units; aux gas flow, 13 arbitrary units; sweep gas flow, 3 arbitrary units; capillary temperature, 260 °C; aux gas heater temperature, 425 °C; spray voltage, 3.6 kV; polarity, positive; MS1 mass range, *m*/*z* 60–900; MS1 resolving power, 35,000 FWHM; the number of data-dependent scans per cycle, 3; MS/MS resolving power, 17,500 FWHM; stepped normalized collision energies, 20, 30, and 40% [52]. For HILIC-MS with the post-column addition of D_2_O, the tubing from the HILIC column and the infusion pump (Fusion 100, Chemyx, Stafford, TX, USA) introducing the D_2_O were coupled with a Tee unit before directing the flow into an ion source.

### 3.5. Data Processing

The HILIC-MS instrumental files from the metabolomic profiling were processed using MS-DIAL software v. 4.92, RIKEN, Yokohama, Japan [62] with the following parameters: (i) data collection: MS1 tolerance, 0.005; MS2 tolerance, 0.01; (ii) peak detection: minimum peak height, 20,000; mass slice width, 0.05; smoothing method, linear weighted moving average; smoothing level, 3; (iii) MS/MS identification settings: accurate mass tolerance (MS1), 0.005; accurate mass tolerance (MS2), 0.005; identification score cut-off, 80%; (iv) alignment: retention time tolerance, 0.05 min; MS1 tolerance, 0.01 Da; peak count filter, 5%; gap filling by compulsion, true. An in-house retention time–*m*/*z* library and MS/MS libraries from various sources (NIST 23, MoNA, and LipidBlast) were used for the initial metabolite annotation. For data processing of the HILIC-MS data with the HDX experiments, MRMPROBS software 2.60, RIKEN [63] was used to obtain the peak heights of particular ions with the following parameters: smoothing method, linear weighted moving average; smoothing level, 3; minimum peak height, 100; retention time tolerance, 0.1 min; amplitude tolerance, 15%; minimum posterior, 70%; MS1 tolerance, 0.002. For structure elucidation, MS-FINDER software v. 3.60, RIKEN [27] was used with the following parameters: mass tolerance (MS1), 0.005 Da; mass tolerance (MS2), 0.005; relative abundance cut-off, 0.1%; LEWIS and SENIOR check, checked; isotopic ratio tolerance, 20%; element ratio check, common range (99.7%); element selection, O, N, P, S; tree depth, 2; local databases + MiNEs + PubChem, checked.

### 3.6. Statistical Analysis

The peak height intensities of *N*^1^-acetylspermidine from the HILIC-MS profiling were normalized to the amount (mg) and log_10_-transformed before processing using GraphPad Prism software v. 10.1, Boston, MA, USA to compare groups (two-way ANOVA, Tukey’s); *p* < 0.05 was considered significant.

## 4. Conclusions

We evaluated the potential of LC-HDX-MS in untargeted metabolomics. The post-column addition of D_2_O in HILIC-MS exhibited limitations, particularly in achieving complete H/D exchange for the polar metabolites. However, the partial and full HILIC-HDX-MS setups, where both mobile phases included D_2_O, demonstrated improved performance with complete H/D exchange for the evaluated metabolites. Applying HILIC-HDX-MS to the unknown metabolite, *N*^1^-acetylspermidine, showcased its potential in reducing false positive annotations. LC-HDX-MS, combined with in silico fragmentation software, effectively reduces potential candidates for structure elucidation and has the potential for diverse untargeted metabolomics studies with specific applications in investigating diseases, circadian rhythms, nutrition, and other areas.

## Figures and Tables

**Table 1:** Overview of the key characteristics of evaluated HILIC-MS methods.

Specification	HILIC-MS	HILIC-MS with D_2_O Co-Infusion	Partial HILIC-HDX-MS	Full HILIC-HDX-MS
Mobile Phase A	H_2_O 10 mM Ammonium Formate 0.125% Formic Acid	H_2_O 10 mM Ammonium Formate 0.125% Formic Acid	D_2_O 10 mM Ammonium Formate 0.125% Formic Acid	D_2_O 10 mM D_5_-Ammonium Formate 0.125% D_2_-Formic Acid
Mobile Phase B	95% Acetonitrile/5% H_2_O 10 mM Ammonium Formate 0.125% Formic Acid	95% Acetonitrile/5% H_2_O 10 mM Ammonium Formate 0.125% Formic Acid	95% Acetonitrile/5% D_2_O 7.5 mM Ammonium Formate 0.125% Formic Acid	95% Acetonitrile/5% D_2_O 7.5 mM D_5_-Ammonium Formate 0.125% D_2_-Formic Acid
Resuspension Solvent	Acetonitrile/H_2_O (4:1, *v/v*) with CUDA, Val-Tyr-Val (IS)	Acetonitrile/H_2_O (4:1, *v/v*) with CUDA, Val-Tyr-Val (IS)	Acetonitrile/D_2_O (4:1, *v/v*) with CUDA, Val-Tyr-Val (IS)	Acetonitrile/D_2_O (4:1, *v/v*) with CUDA, Val-Tyr-Val (IS)
Column Flow Rate	0.4 mL/min	0.4 mL/min	0.4 mL/min	0.4 mL/min
Co-Infusion Solvent	—	D_2_O	—	—
Co-Infusion Flow Rate	—	0.05, 0.1 mL/min	—	—

## Data Availability

The data are contained within this article and in the Supplementary Materials.

## Supplementary Materials

The supporting information can be downloaded at https://www.mdpi.com/article/10.3390/ijms25052899/s1.
