# Supplementary material for: Hydrophilic Interaction Liquid Chromatography–Hydrogen/Deuterium Exchange–Mass Spectrometry (HILIC-HDX-MS) for Untargeted Metabolomics
Source: Int J Mol Sci. 2024 Mar 1;25(5):2899. doi: 10.3390/ijms25052899 (PMC10932214; doi:10.3390/ijms25052899)
Supplement: Supplementary file 1 [file ijms-25-02899-s001.zip › ijms-2849541_Supplementary_Materials_Figures.pdf]

International Journal of *Molecular Sciences*

MDPI

Supplementary Materials

Hydrophilic Interaction Liquid Chromatography–Hydrogen/Deuterium Exchange–Mass Spectrometry (HILIC-HDX-MS) for Untargeted Metabolomics

Tomas Cajka [1,*], Jiri Hricko [1], Stanislava Rakusanova [1], Kristyna Brejchova [1], Michaela Novakova [1], Lucie Rudl Kulhava [1], Veronika Hola [1], Michaela Paucova [1], Oliver Fiehn [2], Ondrej Kuda [1]

[1] Institute of Physiology of the Czech Academy of Sciences, Videnska 1083, 14200 Prague, Czech Republic

[2] West Coast Metabolomics Center, University of California, Davis, 451 Health Sciences Drive, Davis, CA 95616, USA

* Correspondence: tomas.cajka@fgu.cas.cz

Figure S1. Extracted ion chromatograms of an unknown metabolite (4.3 min; *m*/*z* 188.1757) detected in different biological matrices and of different origins (rat, mouse, human).

Figure S2. Extracted ion chromatograms (EICs) and MS1 and MS/MS spectra of N^1-acetylspermidine in rat feces under conventional HILIC-MS and full HILIC-HDX-MS conditions. In HILIC-MS, the EIC at *m*/*z* 188.1757, corresponding to $[M(H_4)+H]^+$, is displayed. Conversely, in HILIC-HDX-MS, the EIC at *m*/*z* 193.2071, corresponding to $[M(D_4)+D]^+$, is shown. MS/MS spectra were acquired at stepped normalized collision energies of 20, 30, and 40%.

Figure S3. Extracted ion chromatograms (EICs) and MS1 and MS/MS spectra of N^1-acetylspermidine in human plasma (NIST SRM 1950) under conventional HILIC-MS and full HILIC-HDX-MS conditions. In HILIC-MS, the EIC at *m*/*z* 188.1757, corresponding to $[M(H_4)+H]^+$, is displayed. Conversely, in HILIC-HDX-MS, the EIC at *m*/*z* 193.2071, corresponding to $[M(D_4)+D]^+$, is shown. MS/MS spectra were acquired at stepped normalized collision energies of 20, 30, and 40%.

Figure S4. MS/MS spectra of (**a**) unknown metabolite (precursor ion *m*/*z* 188.1757) in mouse feces extract, (**b**) N^1-acetylspermidine standard (precursor ion *m*/*z* 188.1757), and (**c**) N^8-acetylspermidine standard (precursor ion *m*/*z* 188.1757). MS/MS spectra were acquired at stepped normalized collision energies of 20, 30, and 40%.

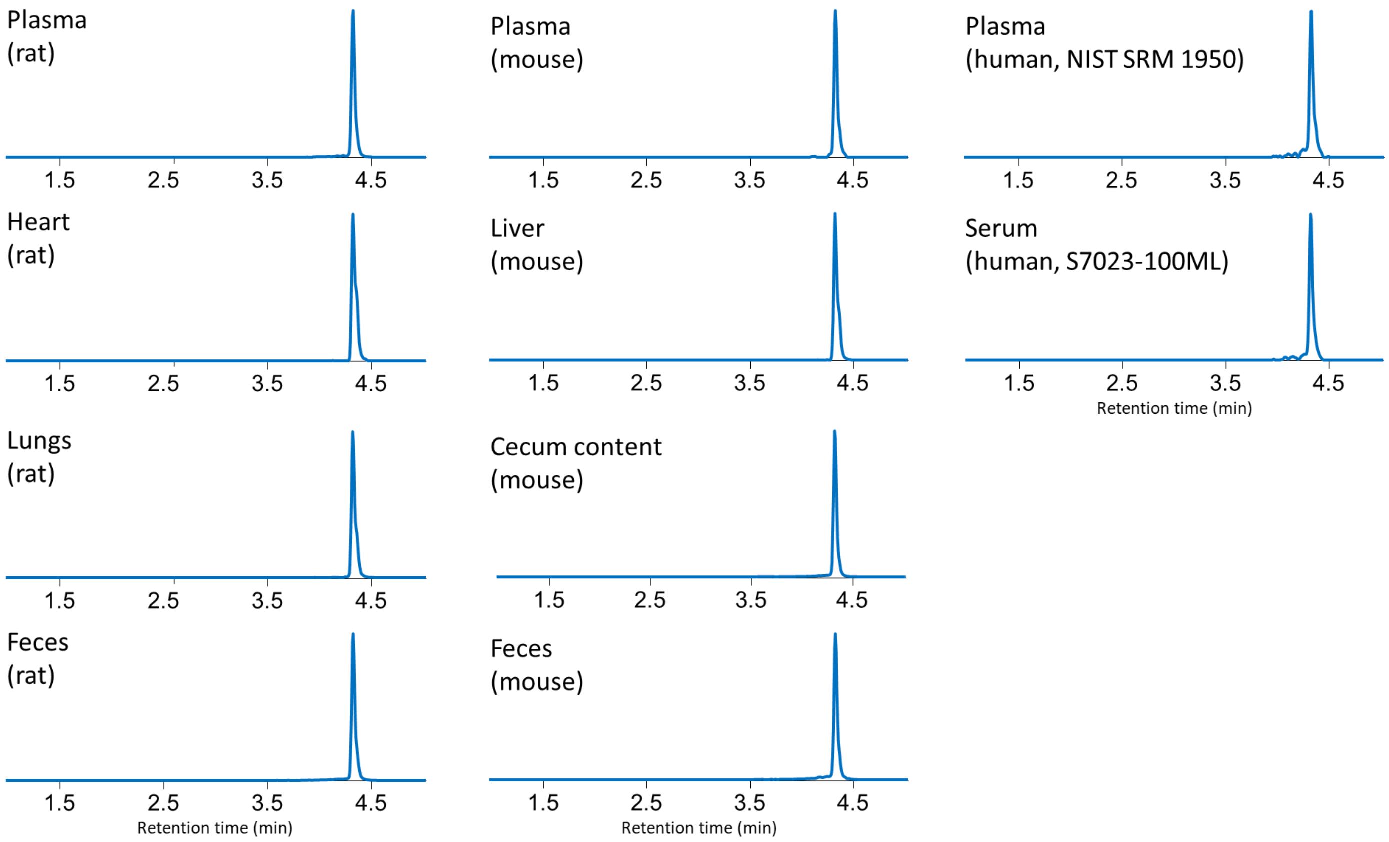

Figure S1. Extracted ion chromatograms of an unknown metabolite (4.3 min; *m*/*z* 188.1757) detected in different biological matrices and of different origins (rat, mouse, human).

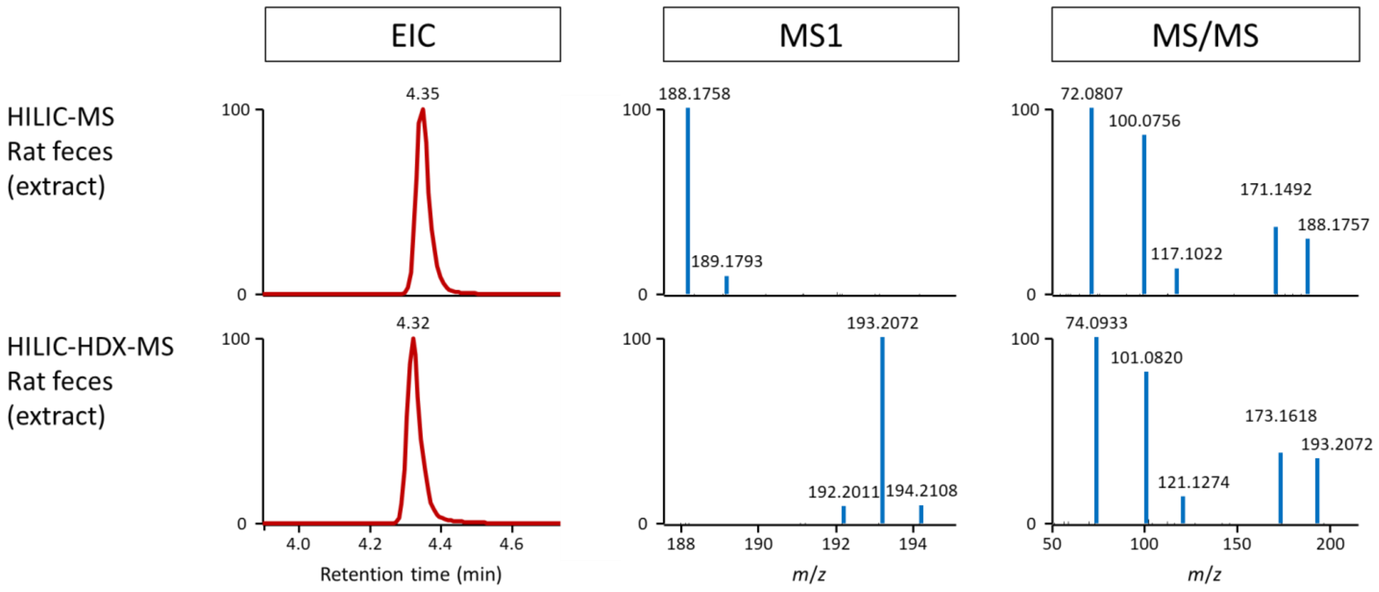

Figure S2. Extracted ion chromatograms (EICs) and MS1 and MS/MS spectra of N^1-acetylspermidine in rat feces under conventional HILIC-MS and full HILIC-HDX-MS conditions. In HILIC-MS, the EIC at *m/z* 188.1757, corresponding to $[M(H_4)+H]^+$, is displayed. Conversely, in HILIC-HDX-MS, the EIC at *m/z* 193.2071, corresponding to $[M(D_4)+D]^+$, is shown. MS/MS spectra were acquired at stepped normalized collision energies of 20, 30, and 40%.

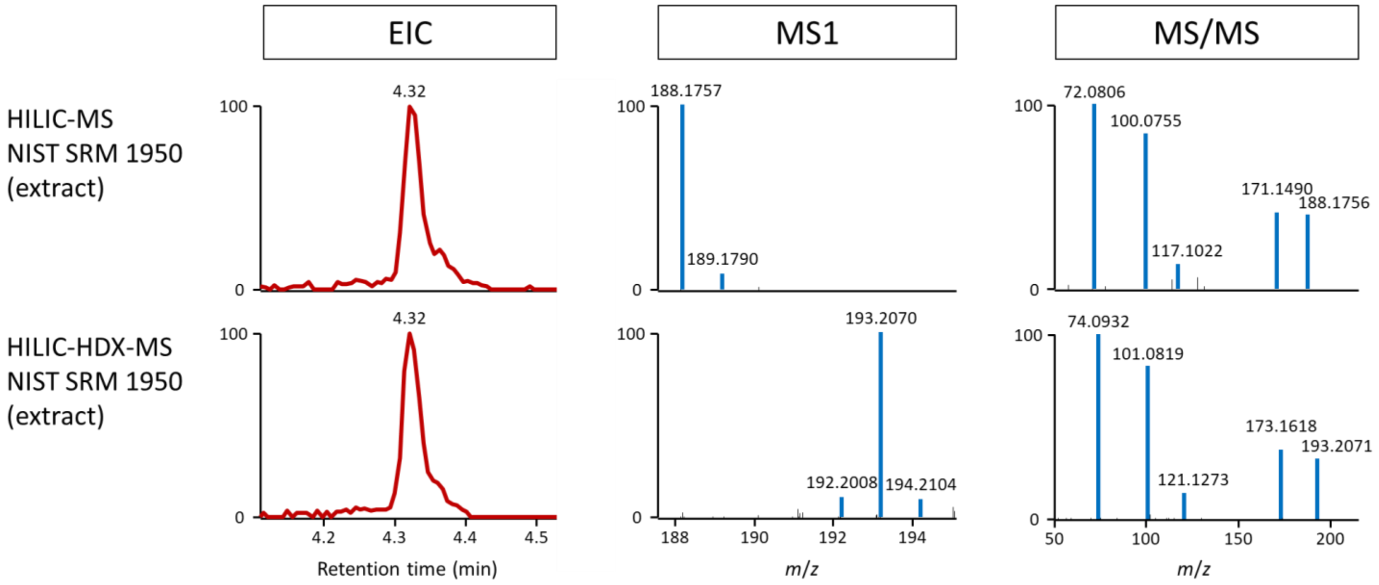

Figure S3. Extracted ion chromatograms (EICs) and MS1 and MS/MS spectra of *N*[1]-acetylspermidine in human plasma (NIST SRM 1950) under conventional HILIC-MS and full HILIC-HDX-MS conditions. In HILIC-MS, the EIC at *m/z* 188.1757, corresponding to $[M(H_4)+H]^+$, is displayed. Conversely, in HILIC-HDX-MS, the EIC at *m/z* 193.2071, corresponding to $[M(D_4)+D]^+$, is shown. MS/MS spectra were acquired at stepped normalized collision energies of 20, 30, and 40%.

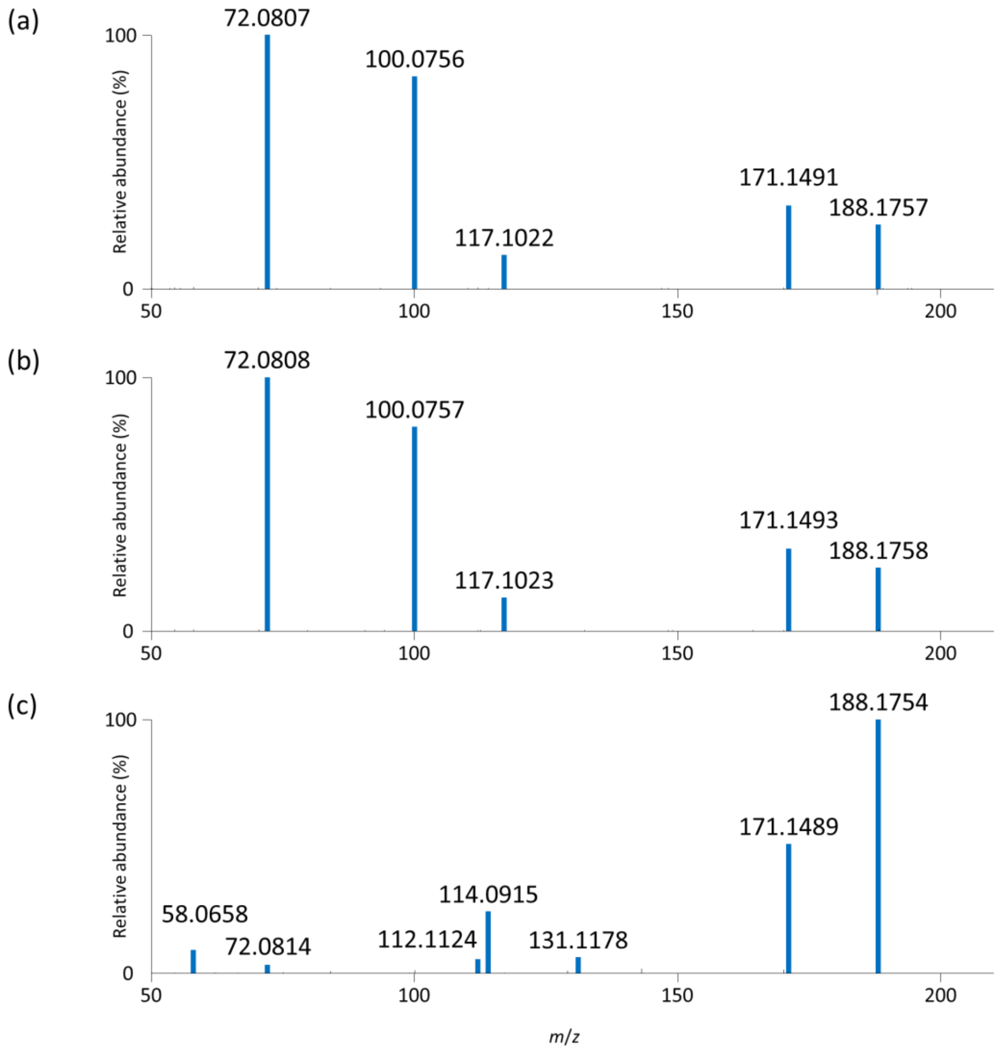

Figure S4. MS/MS spectra of (**a**) unknown metabolite (precursor ion *m/z* 188.1757) in mouse feces extract, (**b**) N^1-acetylspermidine standard (precursor ion *m/z* 188.1757), and (**c**) N^8-acetylspermidine standard (precursor ion *m/z* 188.1757). MS/MS spectra were acquired at stepped normalized collision energies of 20, 30, and 40%.
